# Influence of rubber’s viscoelasticity and damping on vertical dynamic stiffness of air spring

**DOI:** 10.1038/s41598-023-36904-9

**Published:** 2023-06-19

**Authors:** Yinghao Hu, Jianhong Zhang, Jiangqi Long

**Affiliations:** 1 School of Mechanical Electrical Engineering, Wenzhou University, Wenzhou, 325035 China; 2 Zhe Jiang Sensen Auto Parts LTD, Wenzhou, 325204 China

**Keywords:** Mechanical engineering, Nonlinear phenomena

## Abstract

Using the diaphragm-type air spring as the research object. The ratio of the vertical stiffness change caused by compressed air to the total vertical stiffness change was calculated, and it was determined that the nonlinearity of air spring vertical stiffness was mainly caused by the deformation stiffness of the rubber airbag. The variation law of vertical dynamic stiffness of air spring was predicted by theory: due to the material's viscoelasticity, the vertical dynamic stiffness rises as the excitation frequency rises, and the vertical dynamic stiffness decreases with the increase of excitation amplitude due to the damping of the material. An air spring finite element analysis (FEA) and experiment were conducted. The results show that the vertical dynamic stiffness obtained through simulation and experiment is consistent with the theoretical prediction, when various factors such as material nonlinearity, element coupling, and stiffness value sensitivity were considered. This proves that the predicted vertical dynamic stiffness variation law is reliable. The vertical dynamic stiffness obtained from both simulation and experiment showed a strong correlation in numerical values, which verified the accuracy of the FEA model of air spring established in this paper.

## Introduction

Due to its reasonable nonlinear elastic properties, low vibration frequency, effective energy absorption rate, adjustable stiffness characteristics for optimum driving comfort, and other benefits, air springs are popular among automakers and consumers. Air suspension is available on vehicles like the NIO ES8, Audi A8, Hong Qi HS7, and others. In recent years, the vehicle industry has also become quite interested in the research on air springs.

Compressed air stiffness and rubber deformation stiffness make up the majority of air spring stiffness. At the moment, no consistent conclusion has been reached as to whether the main factor influencing air spring stiffness is the stiffness of compressed air^1^ or the deformation stiffness of rubber airbags^2^. In this study, it is concluded that the main factor influencing the stiffness of a diaphragm-type air spring is the deformation stiffness of the rubber airbag by calculating the ratio of compressed air stiffness to total stiffness. The deformation stiffness of rubber airbags is related to rubber material properties such as density, hyperelasticity viscoelasticity, and damping. All these rubber material properties should be considered in the analysis process to obtain reliable results. The analysis of the air spring consists of three different routes, namely the theoretical study, the FEA, and the experimental study. Among them, properties of rubber are involved in theory and FEA studies.

In the theoretical analysis of air spring, rubber damping, and viscoelasticity were the major factors. A study has presented a dynamic stiffness mechanical model of air springs that takes into equivalent damping and hysteresis properties in the theoretical study of air springs^3^. A mechanical model of an air spring with nonlinear properties was developed based on the damping and viscoelasticity of rubber. Additionally, a six-step approach for identifying rubber parameters was suggested^2^.

The influence of rubber damping, and viscoelasticity is also considered in the FEA of rubber products or objects with simple harmonic motion. After setting Rayleigh damping, the displacement result of simple harmonic vibration is more accurate^4^. If the stiffness-proportional damping is not taken into account in the Abaqus/Explicit analysis, high-frequency oscillations will result^5^. The damping of the rubber support ring is used as feedback to analyze the vibration characteristics of the vehicle, and the riding comfort of the vehicle can be improved by changing the parameters related to the rubber damping^6^. The viscoelastic natural frequency of rubber is closer to the experiment results in the finite element modal analysis (FEMA) than the results without viscoelastic^7^. The viscoelasticity of materials affects the stress distribution and overall hysteresis performance of rubber in the relaxation process^8^. The maximum contact pressure and von Mises stress reduced in the simulation results under the rubber hyperelastic-viscoelastic constitutive model compared to the rubber hyperelastic constitutive model, while the contact width increased^9^.

The air spring is made of rubber and primarily performs harmonic motion while working. However, the current FEA of the air spring focuses on the effect of the fabric layer arrangement, initial internal pressure, excitation, and other parameters on the stiffness of the air spring^10–16^. In these studies, only the density and hyperelasticity are considered about rubber. Two important parameters, viscoelasticity and damping are missing.

## Methods

The influence of compressed air and rubber airbag deformation on the stiffness of an air spring was discussed in this paper by combining theory, FEA, and experiment. The goal of study is lowering the forward design cost. Reveal the mechanism of vertical dynamic stiffness of an air spring while. In the following text, stiffness refers to vertical stiffness.

### Influencing factors of air spring stiffness

**Figure 1:**
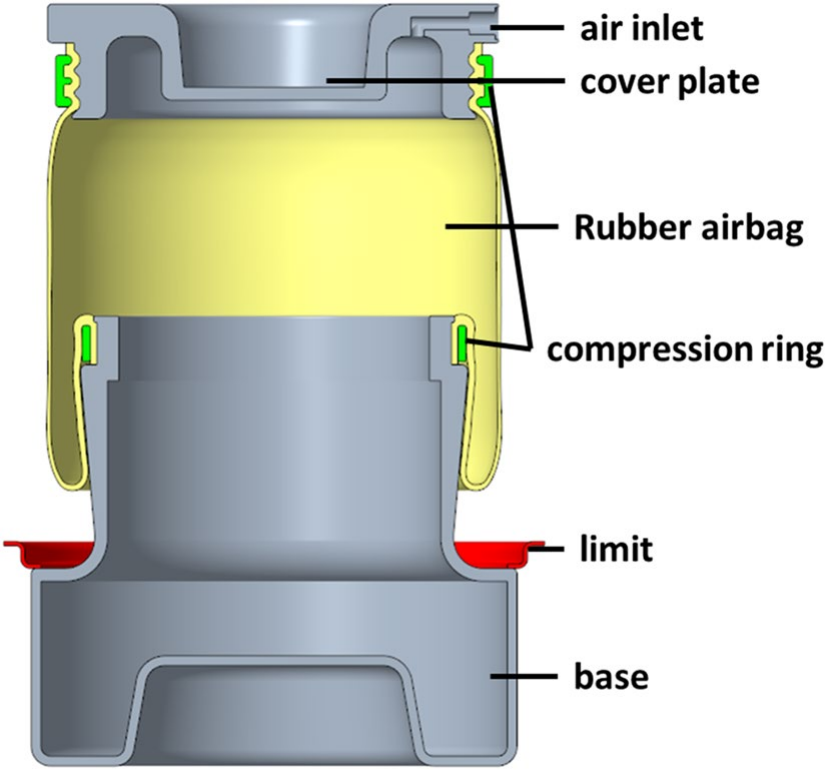
The sectional view of air spring sample structure.

There are two types of air springs: diaphragm and bellows types. The diaphragm-type air spring was focused in this study. Through the cover plate, base, and rubber airbag, the air spring creates a pressurized air column, and then sealed by the pressure ring. Figure 1 depicts the air spring sample that was used in this study.

The air spring has a better force curve than that of a metal one, as evidenced by the fact that during compression, the gas volume of the spring drops and its stiffness improves due to changes in the effective area of the compressed air column and the deformation stress on the rubber airbag. The stiffness of the air spring can be written as:

1 $$ K = K_{a} + K_{r} $$

where *K* is the stiffness of air spring, *K*_*a*_ is the stiffness of compressed air, *K*_*r*_ is the deformation stiffness of rubber airbag.

The stiffness of compressed air *K*_*a*_ can be obtained by taking the derivative of load force with respect to displacement:

2 $$ K_{a} = \dot{A}\left[ {P_{0} \left( {\frac{{V_{0} }}{V}} \right)^{a} - P_{atm} } \right] + \dot{V}\left[ {\frac{{aAP_{0} V_{0}^{a} }}{{V^{a + 1} }}} \right] $$

where *x* is the vertical displacement of the base, *A* is the effective area of the air spring, $\dot{A}$ is the *A* with respect to the displacement, *V* is the volume of air spring at any height, $\dot{V}$ is the *V* with respect to the displacement, *P* is the airbag pressure (absolute air pressure) inside, *P*_*atm*_ is standard atmospheric pressure, *P*_0_ is the internal pressure (absolute pressure) of the air spring, *V*_0_ is the initial volume of air spring, *a* is the gas variability index, for adiabatic and isothermal processes a is 1.4 and 1.0, respectively.

In the static experiment, the change of gas state in the air spring is close to the isothermal process, Therefore, *a* = 1.0 was taken to be the input for the calculation. The effective area of a diaphragm-type air spring does not change significantly during compression and extension^17^. Thus, *A* is a constant, i.e., $\dot{A} = 0$. As this time the rate of change in volume $\dot{V} = A$. The stiffness of compressed air can be expressed as:

3 $$ K_{a} = \frac{{A^{2} P_{0} V_{0} }}{{V^{2} }} $$

**Figure 2:**
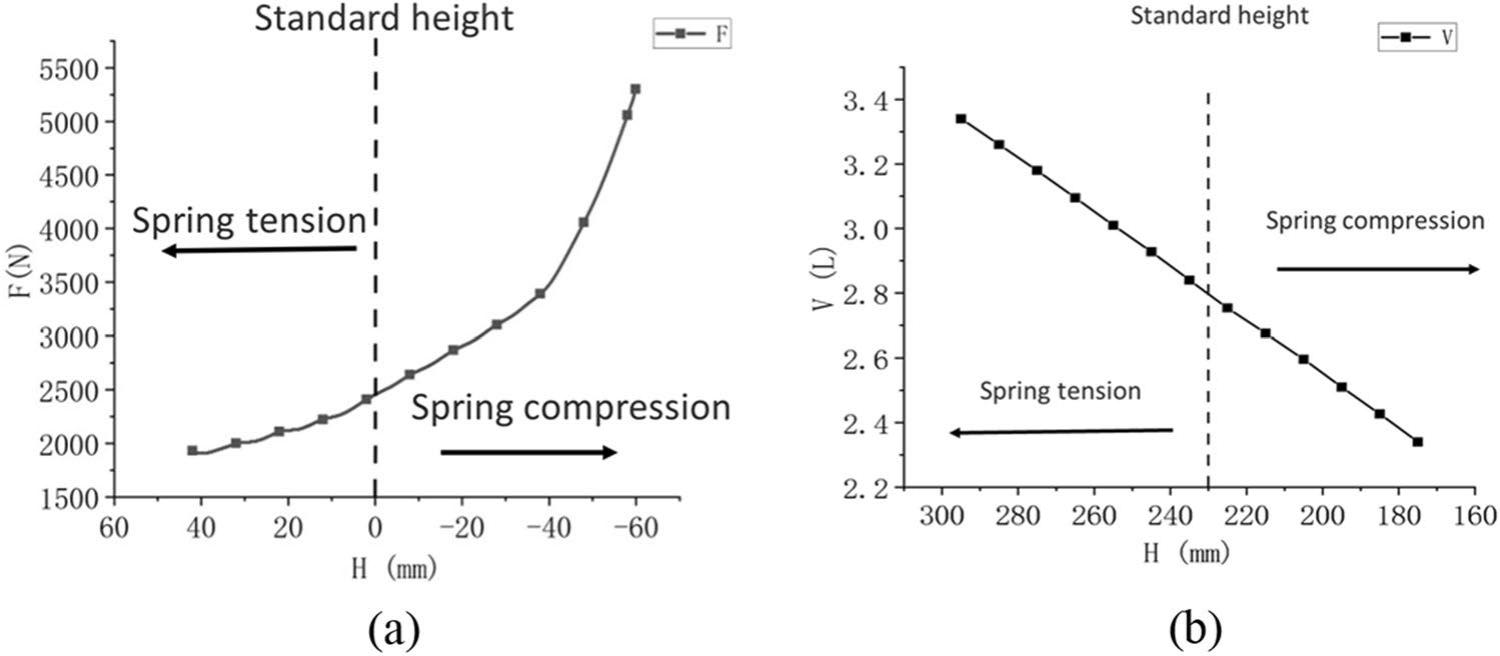
Force–height (F–H) (**a**) and volume–height (V–H) (**b**) curve of the air spring.

According to Eq. (3), the stiffness of compressed air is only related to its gas volume. Static tension and compression experiments and volume experiments were carried out on the samples with an initial pressure of 0.2 MPa, and the experiment data were sorted out in Fig. 2.

Where H is the overall height, F is the reaction force, V is the volume.

According to the experiment data, when the air spring is compressed by 1 mm, the volume of the prototype changes to 0.0083L, which is about 0.29% of the total volume (2.84L) at the standard height. By obtaining the slope of the F–H curve, the spring stiffness was calculated to be 25N/mm and 50N/mm at the original height and the height that was compressed 40 mm, respectively. was calculated as 31.992N/mm using Eq. (6). The stiffness change caused by compressed air only accounted for 27.97% of the total stiffness change in the 0–40 mm process, indicating that the nonlinearity of air spring stiffness was primarily caused by the deformation stiffness of the rubber airbag *K*_*r*_. When the stiffness nonlinearity of the air spring is not considered, the compression amount of the air spring with the same pressure corresponds to the stiffness value one by one. In practical research, it has been found that the stiffness value of the same compression amount changes with the amplitude and frequency of the excitation, that is, the dynamic stiffness changes with the variation of the excitation. In this paper, the influence of the material characteristics of rubber airbag on the dynamic stiffness of air spring and the change law of dynamic stiffness with excitation will be studied.

### Theoretical analysis

Rubber airbag deformation stress is primarily affected by density, hyperelasticity, viscoelasticity, and damping, with density and hyperelasticity affecting static stiffness and viscoelasticity and damping affecting dynamic stiffness. This paper investigates the effect of viscoelasticity and damping on the dynamic stiffness of the air spring.

#### Viscoelasticity

**Figure 3:**
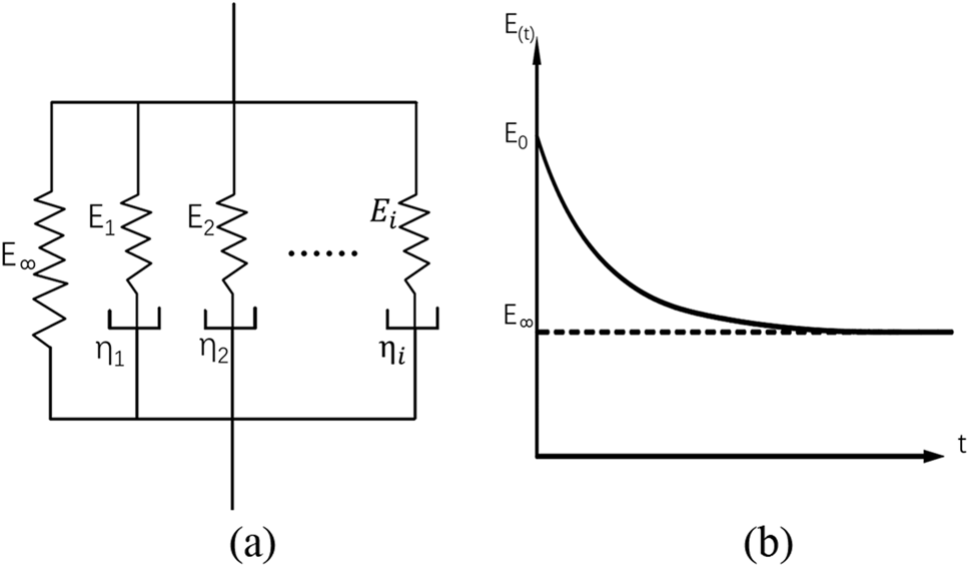
Generalized Maxwell mechanical model (**a**) and generalized Maxwell stress relaxation curve (**b**).

Creep and stress relaxation are properties of viscoelastic materials. Creep is a phenomenon that occurs when a constant stress is applied to a viscoelastic sample and the strain increases with time. Stress relaxation is a phenomenon that keeps the strain constant, and the stress gradually decays with time. When dynamic loads are applied, these two properties are the primary causes of frequency dependence and hysteresis in air springs. The generalized Maxwell model is used to describe the viscoelasticity of rubber in this paper. As shown in Fig. 3, the generalized Maxwell model is made up of multiple Maxwell models and a balanced elastic element *E*_*∞*_ running in parallel.

The overall relaxation modulus can be expressed as^18^:

4 $$ E_{\left( t \right)} = E_{\infty } + \mathop {\mathop \sum \limits_{i = 1} }\limits^{n} E_{i} exp\left( { - \frac{t}{{\tau_{i} }}} \right) $$

where *E*_(*t*)_ is the overall elastic modulus of the generalized Maxwell model at time t, *E*_*∞*_ is the balance elastic, *E*_*i*_ and *η*_*i*_ are elastic modulus and damping of the *i*th Maxwell model, respectively, $\tau_{i} = \eta_{i} /E_{i}$. When *t* = 0, $e_{i} = E_{i} /E_{0}$ is introduced, and Eq. (4) is transformed into Prony series:

5 $$ E_{\left( t \right)} = E_{0} \left[ {e_{\infty } + \mathop {\mathop \sum \limits_{i = 1} }\limits^{n} e_{i} exp\left( { - \frac{t}{{\tau_{i} }}} \right)} \right] $$

Equation (5) is the long-term elastic modulus form, corresponding to the generalized Maxwell model shown in Fig. 3a.

6 $$ E_{\left( t \right)} = E_{0} \left[ {1 - \mathop {\mathop \sum \limits_{i = 1} }\limits^{n} e_{i} exp\left( { - \frac{t}{{\tau_{i} }}} \right)} \right] $$

Equation (6) is the transient elastic modulus form, corresponding to the stress relaxation curve shown in Fig. 3b. Similarly, the material's shear modulus *G*_(*t*)_ and volume modulus *κ*_(*t*)_ are expressed as follows:

7 $$ G_{\left( t \right)} = G_{0} \left[ {1 - \mathop {\mathop \sum \limits_{i = 1} }\limits^{n} g_{i} exp\left( { - \frac{t}{{\tau_{i}^{G} }}} \right)} \right] $$

8 $$ \kappa_{\left( t \right)} = \kappa_{0} \left[ {1 - \mathop {\mathop \sum \limits_{i = 1} }\limits^{n} k_{i} exp\left( { - \frac{t}{{\tau_{i}^{\kappa } }}} \right)} \right] $$

The excitation variation is primarily reflected in the frequency and amplitude in the dynamic stiffness FEA of air spring. Equation (16) can be used to define the viscoelasticity of the rubber material. Rubber is an incompressible material, *κ*_(*t*)_ = 0. Take the Fourier transform of Eq. (7):

9 $$ G_{S\left( \omega \right)} = G_{0} \left( {1 - \mathop {\mathop \sum \limits_{i = 1} }\limits^{N} g_{i} } \right) + G_{0} \mathop {\mathop \sum \limits_{i = 1} }\limits^{N} \frac{{g_{i} \tau_{i}^{2} \omega^{2} }}{{1 + \tau_{i}^{2} \omega^{2} }} $$

10 $$ G_{L\left( \omega \right)} = G_{0} \mathop {\mathop \sum \limits_{i = 1} }\limits^{N} \frac{{g_{i} \tau_{i} \omega }}{{1 + \tau_{i}^{2} \omega^{2} }} $$

where *ω* is the angular velocity, *G*_*S*(*ω*)_ is the storage modulus, and *G*_*L*(*ω*)_ is the loss modulus.

According to Eq. (10), as *ω* increases, *G*_*L*(*ω*)_ decreases. The energy dissipation of the rubber material is reduced, which means the stored energy is increased, the overall stiffness of the air spring is improved, that is, with the increase of the excitation angular velocity, the stiffness of the air spring will also be improved.

#### Damping

Rayleigh damping is used in ABAQUS for material damping. Rayleigh damping, including mass damping and stiffness damping, is transformed by the Kelvin-Voigt model through Principle of Virtual Displacement.

The total damping matrix C can be written as^19^:

11 $$ C = \alpha M + \beta S $$

where *α* and *β* are the mass and stiffness damping coefficients, respectively. *M* and *S* are the mass and stiffness damping matrices, respectively. The equivalent stress of nodes corresponding to mass damping is:

12 $$ F_{e} = - \alpha M \cdot \dot{d} $$

where *F*_*e*_ is the equivalent stress of the node, and $\dot{d}$ is the velocity vector at the node.

According to Eq. (12), as the velocity vector of the airbag increases, the damping force generated at the node on the airbag increases, the rubber deformation stress generated at the node decreases, and the stiffness of the overall air spring decreases, as the excitation amplitude increases, the stiffness of the air spring decreases.

At low frequencies, the mass damping component bears the majority of the damping effect. The stiffness damping part is primarily responsible for the damping effect at high frequencies. Stiffness damping in Rayleigh damping reflects the blocking effect of materials on dynamic response, which is related to strain and strain rate. The contribution of strain rate to damping force is ignored in FEA, resulting in some mistakes in stress calculation. The blocking effect of the environment on rubber vibration is reflected by mass damping.

## FEA and experiment

### Dynamic stiffness simulation

ABAQUS has a rich cell library and is well-suited for analyzing highly nonlinear problems. The compressed gas sealed in the airbag is simulated as an ideal gas, and its heat exchange with the outside world during the tension and compression processes is ignored. Non-convergence in the simulation can be caused by the mechanical nonlinear characteristics of the rubber model, the setting of the rebar element used to simulate the fabric layer, the size and quality of the mesh, and other factors. In this paper, the Explicit solver was used to solve the calculation, which consumes more computational resources but was easier to converge. The airbag is connected to the cover plate and the base through a common node method. When creating a rigid body constraint, set the base and limit to a same reference point.

**Figure 4:**
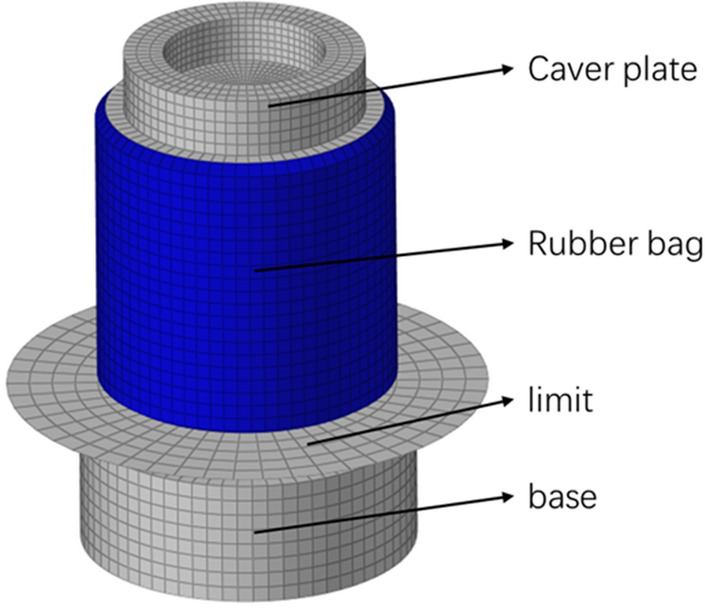
Structure diagram of simulation model.

The cover plate and base of the air spring sample used in this paper contain some gases as well, and the closed fluid cavity's composition is divided into rubber and non-rubber parts. The simulation model of the integration of airbag, cover plate, and base is established to ensure the accuracy of the airbag volume, as shown in Fig. 4.

#### Parameter setting

In ABAQUS, the Mooney-Rivlin model is used to describe the hyperelasticity of rubber materials. The specific expression^20^ is as follows:

13 $$ U = C_{10} \left( {I_{1} - 3} \right) + C_{01} \left( {I_{2} - 3} \right) + \frac{1}{D}(J_{e} - 1)^{2} $$

where *U* is the strain potential energy of rubber, *I*_1_ and *I*_2_ are the first and second partial strain variables respectively, *C*_10_ and *C*_01_ are the hyperelastic material parameters related to hardness, *J*_*e*_ is the elastic volume ratio, and *D* is the volume correlation coefficient.

Acrylonitrile-Butadiene Rubber is frequently used in the manufacture of air springs. The hardness is 60IRHD. According to the rubber hardness, and by comparing the static stiffness test and simulation results of the air spring, determine the hyperelastic parameters of the airbag rubber as *C*_10_ = 1 MPa, *C*_01_ = 0.04 MPa, and *D* is the volume correlation coefficient, and rubber materials are approximately incompressible, so *D* = 0 is taken. In ABAQUS, *D* = 0 indicates that the material is incompressible and will be automatically replaced with a value close to 0 during calculation. Rubber Rayleigh damping *α* = 0.988, *β* = 0.00049 can be found in the manual. Rubber viscoelastic parameters are shown in Table 1^21^.

**Table 1:** Viscoelastic parameters.

	*g*_*i*_	*τ*_*i*_
*i* = 1	1.33E-05	0.665
*i* = 2	0.0157	0.0206
*i* = 3	0.0407	0.0199
*i* = 4	0.813	0.000218

The fabric is made of PA66 material, with elastic modulus *E* = 2000 MPa, Poisson's ratio *μ* = 0.28 and density *ρ* = 1.14 × 10^−9^ ton/mm^3^^10^. See Table 2 for setting of fabric rebar property parameters.

**Table 2:** Fabric parameters.

Layer	Area (mm^2^)	Spacing (mm)	Angle (°)	Position (mm)
1	0.28	1	50	0.5
2	0.28	1	-50	-0.5

#### Interaction settings

Simplify the air spring model. The contact nonlinearity of the air spring in this paper is mainly reflected in the air bag, the base, and the limit. Add surface to surface contact, select non airbag surface as the main contact surface, and airbag surface as the secondary contact surface. The tangential contact method is "penalty", and the friction coefficient is 0.15; The normal direction is "hard" contact, and the default constraint execution method is.

**Figure 5:**
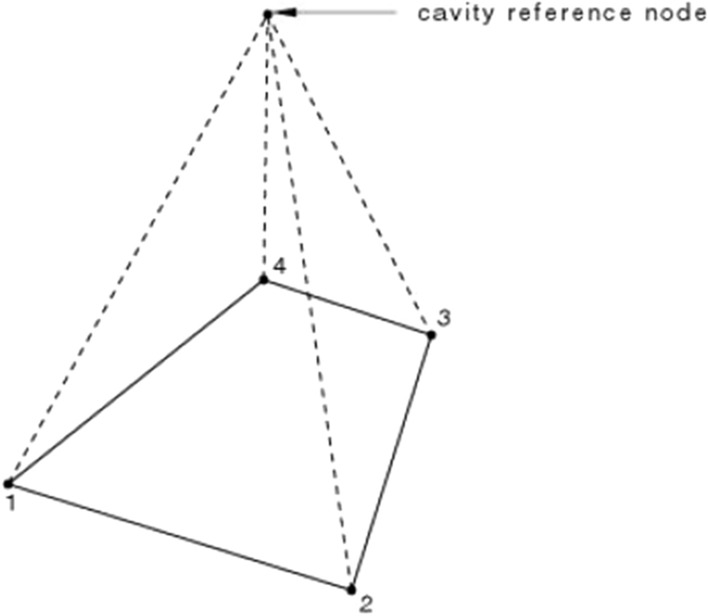
F3D4 unit automatically generated by fluid cavity.

The gas–solid coupling mode of the air spring is simulated by setting the fluid cavity. The surface except the limit is selected as the fluid cavity surfaces. In the experiment, nitrogen is rushed into the air bag. The molecular weight of ideal gas is set to 2.89 × 10^−5^, the molar heat capacity is 37,410, and the ambient pressure is set to 0.1 MPa. Select a point at the midpoint of the airbag on the axis of symmetry as the reference point. ABAQUS will automatically generate F3D3 (triangular shell element) and F3D4 (quadrilateral shell element) elements with reference points and nodes of each shell element contained in the fluid cavity as vertices, as shown in Fig. 5.

#### Analysis step and boundary condition setting

**Figure 6:**
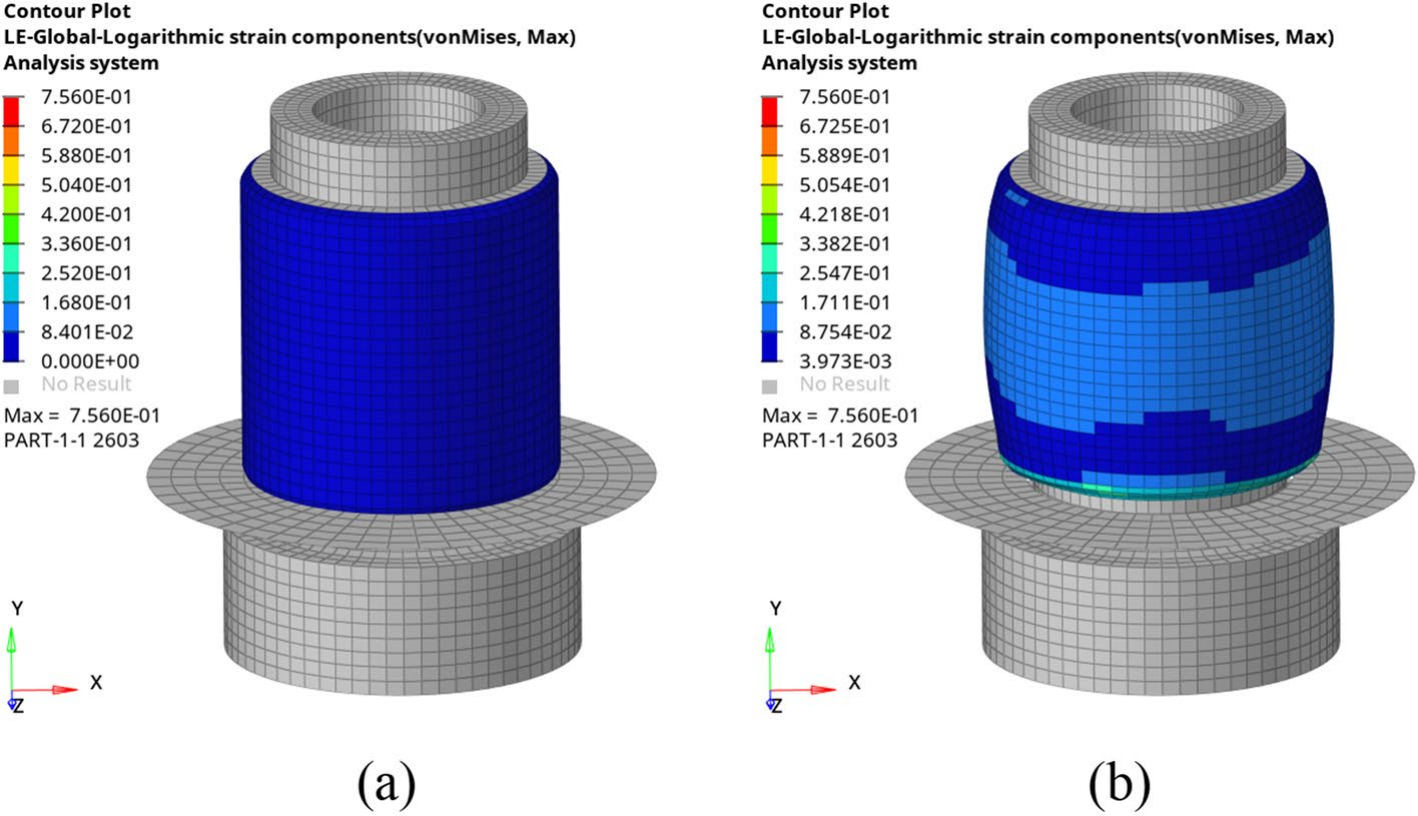
Strain nephogram comparison of before (**a**) and after (**b**) inflation.

In this simulation, one step was set for inflation analysis and ten steps for incentive analysis. The six degrees of freedom (DOF) of the cover plate and the base are constrained during the inflating step, and the air pressure is filled into the fluid cavity. To better reflect the force and changes of the airbag after inflation, a comparison diagram of the simulation model before and after inflation is compiled, as shown in Fig. 6.

The base's movement DOF along the Y-axis is released during the excitation analysis step. Equation (14) depicts the software's specific excitation input:

14 $$ a = A_{0} + \left[ {A\cos \omega \left( {t - t_{0} } \right) + B\sin \omega \left( {t - t_{0} } \right)} \right] $$

where *a* is the excitation input, *A*_0_ is the initial amplitude, *A* and *B* are the amplitudes of cosine and sine function respectively, *ω* is the angular velocity, *t*_0_ is the initial time.

*A*_0_, *A*, *t*_0_ to 0 and *B* was set to 1, and the initial pressure and amplitude are fixed. Each excitation analysis step, corresponding to an excitation frequency of 1–10 Hz, the angular velocity was set to $$\omega = 2*pi*f$$, and submit the calculation. Then the initial pressure was changed, the excitation amplitudes were set to 6 mm, 8 mm, 10 mm, 12 mm, 14 mm respectively, and the simulation was repeated until the simulation calculation was completed for all pressures and amplitudes combinations.

### Dynamic stiffness experiment

**Figure 7:**
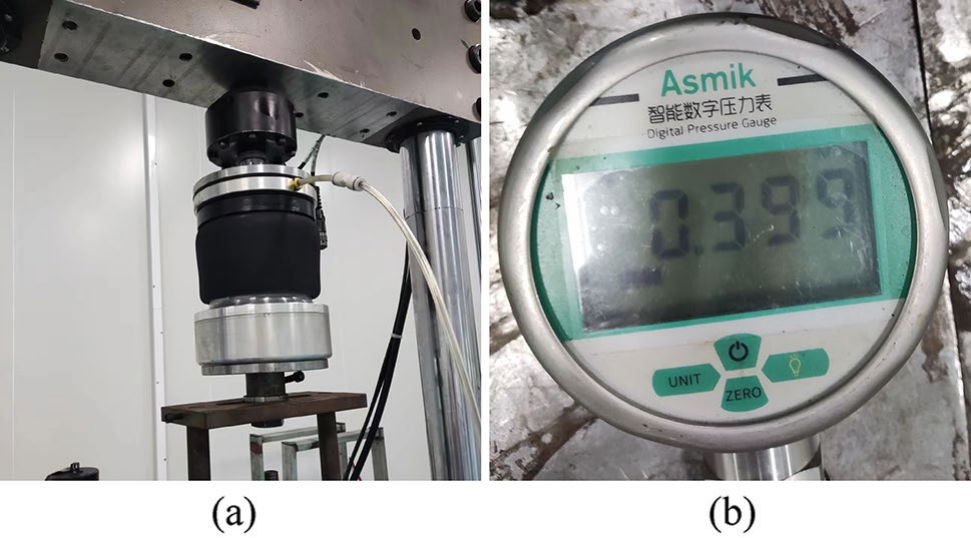
Air tightness test (**a**) and experiment result after 24 h (**b**).

The vertical mechanical properties of a diaphragm-type air spring were tested in accordance with Chinese national standards^22^. The air tightness test was performed first, according to the experiment requirements, and the MTS experimental machine was chosen as the test equipment. Inflate the sample to 0.4 MPa, measure the pressure drop after 24 h, and it is qualified if it is not greater than 0.05 MPa as shown in Fig. 7.

**Figure 8:**
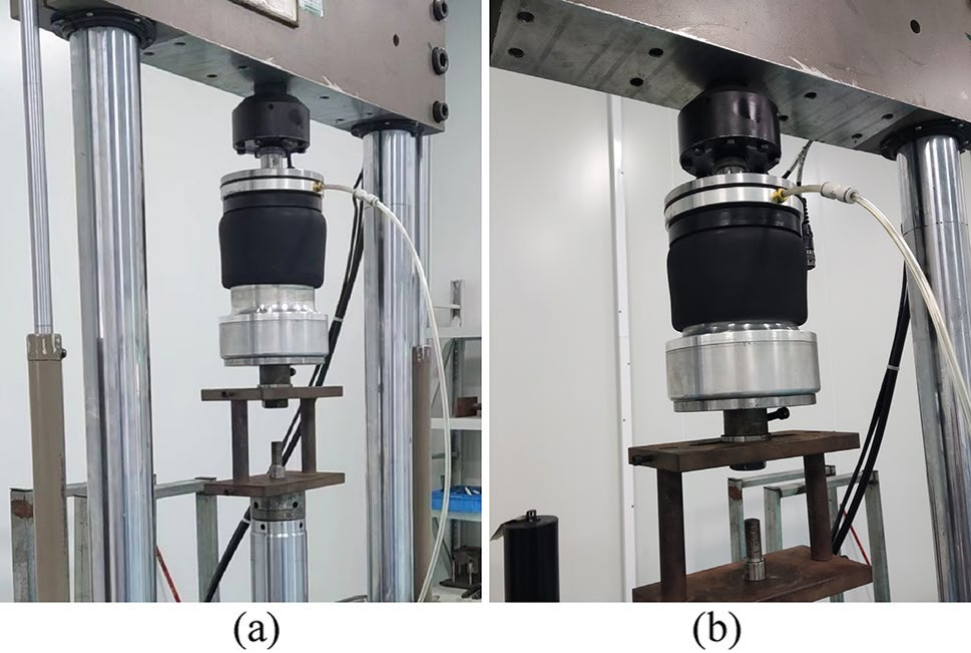
Inflation (**a**) and (**b**) compression test process of air spring.

The stiffness test was performed on a sample with good air tightness. The experimental procedure is as follows: adjust the air spring to the standard height after inflation, and adjust the air pressure to 0.2 MPa, 0.4 MPa and 0.6 MPa respectively. The origin is the base position of the air spring at standard height, the cover plate is fixed, and the excitation amplitudes are 6 mm, 8 mm, 10 mm, 12 mm, 14 mm respectively, and according to the operating frequency range of the car suspension, set the test excitation frequency to 1–10 Hz, with an increment of 1 Hz. Figure 8 shows the inflation and compression process of air spring on MTS.

## Results and discussion

After outputting the time-displacement and time-force data of the base respectively, the relationship between displacement and force is sorted out. The stiffness of the air spring under different excitation is obtained by calculating the slope of the curve. Because there are so many stiffness data, sort out the stiffness value when the air spring height under each excitation is 230 mm (enterprise standard).

### Influence of frequency on the stiffness of air spring

#### Comparison of FEA and theory results

**Figure 9:**
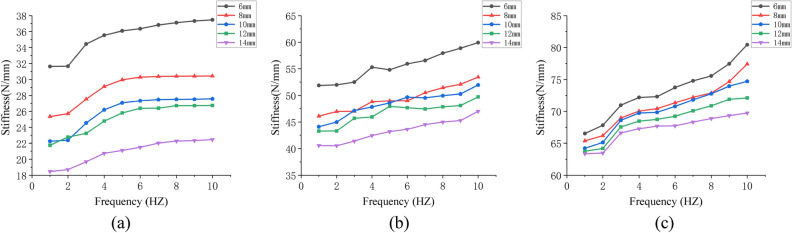
(**a**), (**b**), and (**c**) represent the frequency–stiffness relationship at 0.2 MPa, 0.4 MPa, and 0.6 MPa of simulation.

To sort out the simulation results in the form of curves, use frequency as the abscissa and stiffness as the ordinate, as shown in Fig. 9.

According to the change trend of the curve in Fig. 9, it was discovered that the dynamic stiffness of an air spring increases with frequency under three different air initial pressures. This demonstrates that the simulation results correspond to the theoretical analysis conclusions.

#### Comparison of experiment and theory results

**Figure 10:**
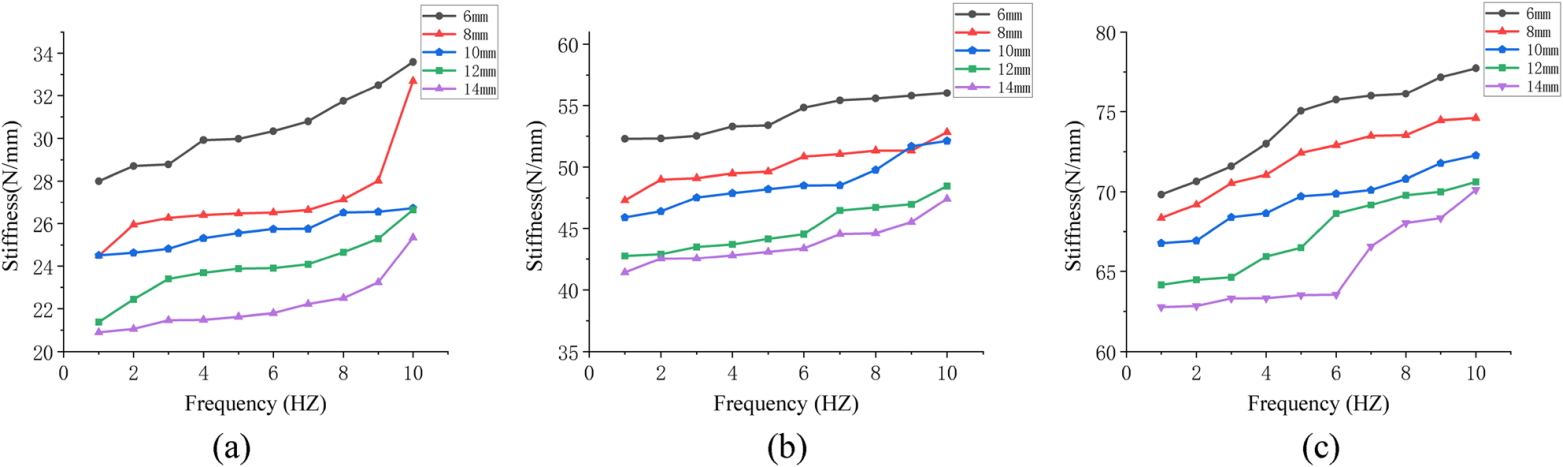
(**a**), (**b**), and (**c**) represent the frequency–stiffness relationship at 0.2 MPa, 0.4 MPa, and 0.6 MPa of experiment.

To sort out the experiment results in the form of a curve, use frequency as the abscissa and stiffness as the ordinate, as shown in Fig. 10.

According to the change trend of the curve in Fig. 10, it was discovered that the dynamic stiffness of an air spring increases with frequency under three different air initial pressures. This demonstrates that the experiment results correspond to the theoretical analysis conclusions.

#### Comparison of FEA and experiment results

In terms of stiffness variation trend and stiffness value, the experimental and simulation results are compared.

**Figure 11:**
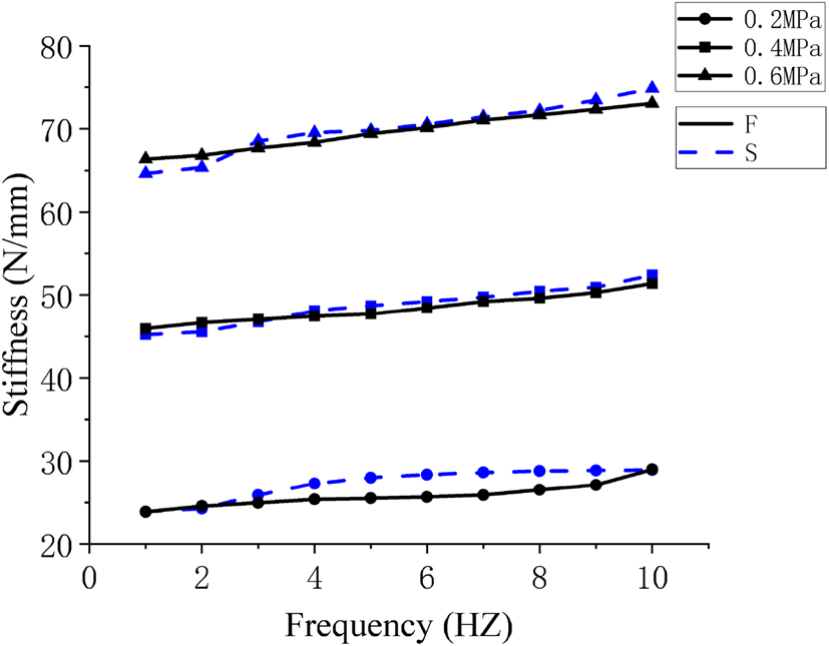
Comparison of frequenc–-stiffness curves between simulation and experiment.

In this paper, five groups of tests of air springs with amplitudes of 6 mm, 8 mm, 10 mm, 12 mm and 14 mm were carried out respectively. Each group of experiments is further divided into ten data sets ranging from 1 to 10HZ. To simplify the data and observe the effect of frequency on stiffness more clearly, a test group data with the amplitude of 10 mm was selected to draw a frequency stiffness curve. Figure 11 depicts the results. The curves F and S represent the simulation and experiment results, respectively.

The data in Fig. 11 show that the change trend of dynamic stiffness is consistent, showing that stiffness increases with frequency increase at 1–10 Hz, what validates the theoretical conclusions once more. In terms of numerical values, there is a small error between the simulation and experimental results. Comparing Figs. 6b and 8a it was found that this may be due to differences in the deformation of the airbag during the simulation process and the deformation of the airbag during the test, resulting in errors in the contact area and contact force.

The Pearson correlation coefficient (PCCs) of calculated experimental and simulation frequency-stiffness data is shown in Table 3.

**Table 3:** PPCs of experimental and simulation data.

Pressure	0.2 (MPa)	0.2 (MPa)	0.2 (MPa)
PPCs	0.87446	0.91341	0.98225

The data in Table 3 show that the dynamic stiffness corresponding to different frequencies under each initial air pressure is very consistent in value, indicating a strong correlation.

### Influence of amplitude on the stiffness of air spring

#### Comparison of FEA and theory results

**Figure 12:**
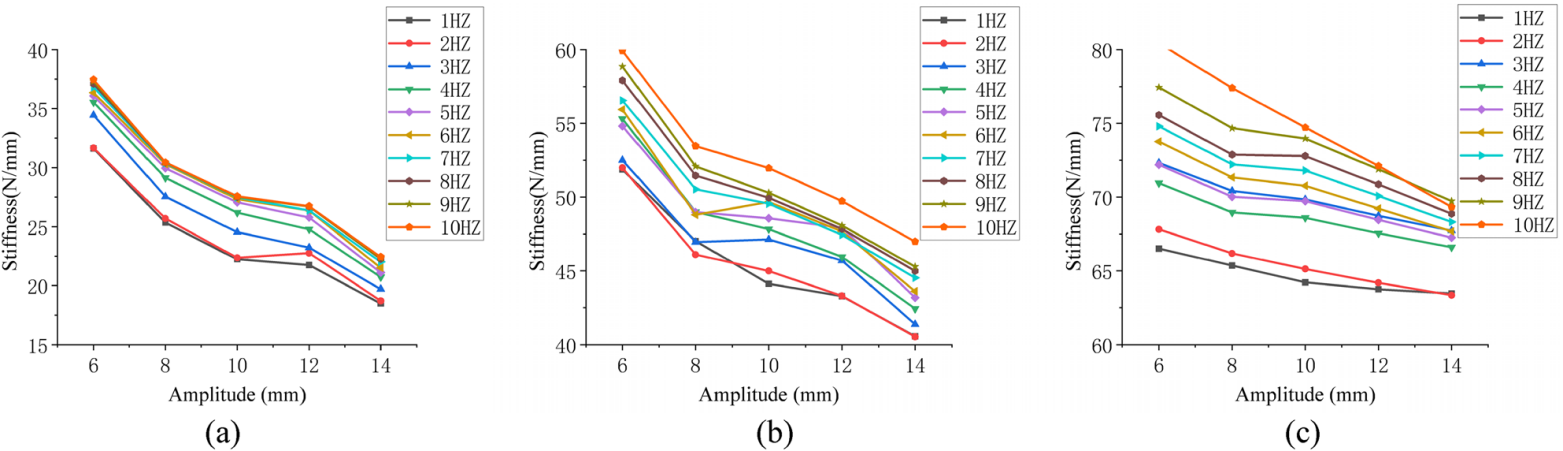
(**a**), (**b**), and (**c**) represent the amplitude–stiffness relationship at 0.2 MPa, 0.4 MPa, and 0.6 MPa of simulation.

To sort out the simulation results in the form of a curve, use amplitude as the abscissa and stiffness as the ordinate, as shown in Fig. 12.

The stiffness of the air spring decreases with increasing amplitude under three different air pressures, according to the change trend of the curves in Fig. 12. This demonstrates that the simulation results correspond to the theoretical analysis conclusions.

#### Comparison of FEA and theory results

**Figure 13:**
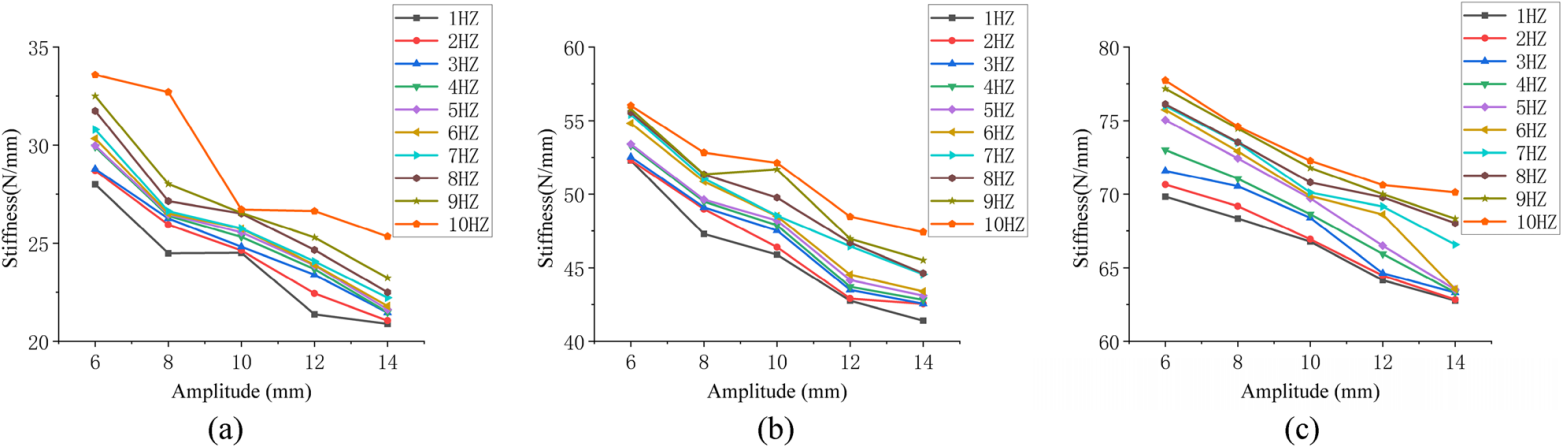
(**a**), (**b**), and (**c**) represent the amplitude–stiffness relationship at 0.2 MPa, 0.4 MPa, and 0.6 MPa of experiment.

To sort out the experiment results in the form of a curve, use amplitude as the abscissa and stiffness as the ordinate, as shown in Fig. 13.

The stiffness of the air spring decreases with increasing amplitude under three different air pressures, according to the change trend of the curves in Fig. 13. This demonstrates that the experiment results correspond to the theoretical analysis conclusions.

#### Comparison of FEA and theory results

**Figure 14:**
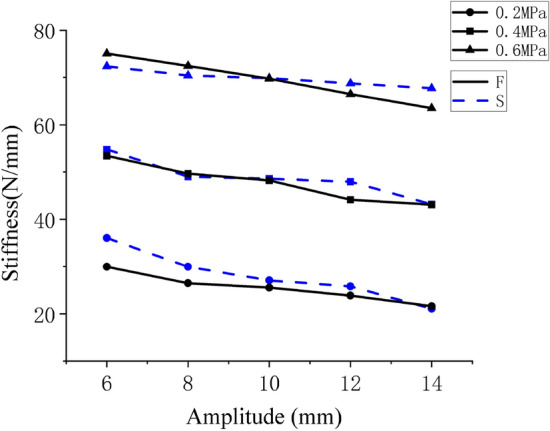
Comparison of amplitude–stiffness curves between simulation and experiment.

Only the amplitude-stiffness curves of simulation and experiment are drawn here when the frequency is 5HZ to simplify the data. In addition, a clear observation of the effects of amplitudes on stiffness can be obtained, Fig. 14 depicts the results. The curves F and S represent the simulation and experiment results, respectively.

The data in Fig. 14 shows that the simulation and experimental stiffness results remain consistent in terms of change trends, with both showing a decrease with increasing amplitude at 6–14 mm. In terms of numerical values, there is a small error between the simulation and experimental results. The possible causes of errors are like those in Fig. 11.

The PCCs of calculated experimental and simulation amplitude-stiffness data is shown in Table 4.

**Table 4:** PPCs of experimental and simulation data.

Pressure	0.2 (MPa)	0.4 (MPa)	0.6 (MPa)
PPCs	0.87446	0.91341	0.98225

The data in Table 4 show that the dynamic stiffness corresponding to different amplitudes under each initial air pressure is very consistent in value, indicating a strong correlation.

As previously stated, the stiffness of an air spring is determined by calculating the slope of the force displacement curve, which is extremely sensitive. The deformation of the airbag shell element and the fabric layer rebar element is inconsistent during the simulation process, and the damping force caused by strain rate is ignored. These factors have little effect on bearing capacity but have a greater impact on stiffness values. The results of the simulation in this paper show a strong correlation with the experimental results, demonstrating that this modeling method with integrated structure and the addition of rubber damping and viscoelastic parameters can better simulate the dynamic stiffness changes of air springs.

## Conclusion

This paper focuses on the diaphragm-type air spring and discusses the effects of rubber damping and viscoelasticity on the air spring's dynamic stiffness. By comparing the stiffness calculated using the formula to the stiffness measured in the experiment, it is determined that the stiffness of the rubber airbag is the primary cause of the air spring's nonlinear stiffness. Fourier transform of Prony series transient modulus form of generalized Maxwell model shows that with the increase of angular velocity, the loss energy decreases, the stored energy increases, and the overall stiffness of air spring increases. By analyzing the Rayleigh damping principle of mass and stiffness damping matrices, it is concluded that the larger the velocity vector of the airbag, the greater the damping force generated at the node on the airbag, the smaller the rubber deformation stress generated at the node, and the smaller the overall stiffness of the air spring. According to the national standard design air spring simulation and experimental conditions, the accuracy is verified.

## Data Availability

All datasets used and/or analyses carried out and results obtained are available from the corresponding author on reasonable request.
